# An Unusual Pattern of Hepatic Involvement in Plasma Cell Leukemia

**DOI:** 10.7759/cureus.104296

**Published:** 2026-02-26

**Authors:** Matt Andrew M Paz, Amira Hamed, Charles Ehster, Robben Schat, Supriya Gupta, Ismail Elbaz Younes, Byoung Uk Park

**Affiliations:** 1 Department of Laboratory Medicine and Pathology, University of Minnesota, Minneapolis, USA; 2 Department of Radiology, University of Minnesota, Minneapolis, USA; 3 Department of Medicine, Division of Hematology, Oncology, and Transplantation, University of Minnesota, Minneapolis, USA

**Keywords:** extramedullary disease, hepatic mass, liver involvement, plasma cell leukemia, plasma cell neoplasm

## Abstract

Plasma cell leukemia (PCL) is a rare and aggressive plasma cell neoplasm associated with dismal outcomes. Organ involvement in PCL, including the spleen and the liver, is rare and commonly manifests as diffuse infiltration of neoplastic plasma cells.

We report a patient with PCL, status post combination chemotherapy, identified to have hepatic lesions on positron emission tomography as part of a pre-bone marrow transplant workup. Biopsy of the hepatic mass revealed sheets of atypical plasma cells within the hepatic parenchyma. Immunohistochemical staining showed strong expression of CD138 and MUM1. In situ hybridization demonstrated lambda light chain restriction, confirming hepatic involvement of the patient’s known PCL. The patient was subsequently recommended for second-line CAR-T therapy in lieu of the planned autologous transplant. This case demonstrates an unusual manifestation of PCL and the importance of maintaining a broad differential diagnosis for hepatic lesions in patients with aggressive plasma cell neoplasms due to its implications for accurate diagnosis and treatment planning.

## Introduction

Plasma cell leukemia (PCL) is a rare, highly aggressive plasma cell neoplasm characterized by circulating clonal plasma cells in the peripheral blood. Although first described in the hematopathology literature over a century ago, it remains an uncommon diagnosis that poses diagnostic and therapeutic challenges [1-3].

Clinically, PCL is subdivided into two distinct entities based on disease presentation. Primary PCL (pPCL) arises de novo in patients without a prior history of multiple myeloma (MM). In contrast, secondary PCL (sPCL) represents a leukemic transformation of relapsed or refractory MM [4-7]. Approximately 60-70% of cases are classified as pPCL, with the remaining representing secondary disease [5,7]. These subtypes differ not only in clinical context but also in disease behavior. pPCL is associated with an aggressive clinical course, poor response to therapy, and dismal prognosis, whereas sPCL typically represents a terminal phase of MM and occurs in approximately 1% of myeloma cases [6-10]. Collectively, PCL accounts for only 2-4% of all plasma cell neoplasms [6-11].

The diagnostic criteria for PCL have evolved over time, and the formal criteria were first established in 1974, when Kyle et al. defined the disease as the presence of more than 20% plasma cells in the peripheral blood and an absolute plasma cell count exceeding 2 × 10⁹/L [3,4,7]. Subsequent reassessment by the International Myeloma Working Group (IMWG) suggested that these criteria may be overly restrictive, proposing that fulfillment of either parameter alone may be sufficient for diagnosis [5,7]. More recently, a 2021 IMWG review of retrospective analyses supported lowering the diagnostic threshold to >5% circulating plasma cells in the peripheral blood, reflecting increased recognition of clinically meaningful disease at lower levels of leukemic involvement [5]. The WHO Revised 4th Edition maintained the traditional diagnostic criteria for PCL (≥20% circulating plasma cells and/or an absolute count ≥2 × 10⁹/L) however, the International Consensus Classification (ICC) and WHO Classification of Haematolymphoid Tumours, 5th Edition (WHO-HAEM5) subsequently adopted a lower threshold of ≥5% clonal circulating plasma cells [12-14].

Plasma cell neoplasms are primarily bone marrow-based malignancies. However, clonal plasma cells may acquire the ability to proliferate outside the marrow microenvironment, resulting in extramedullary involvement, most commonly as mass-forming soft-tissue plasmacytomas [11-17]. Although PCL likewise represents dissemination beyond the marrow, it is classified separately as a distinct leukemic entity and is generally not included under “extramedullary disease” in IMWG frameworks [7,11,15].

Clinically apparent organ involvement in PCL frequently manifests as organomegaly, particularly involving the spleen and liver [3,7-10]. Postmortem examinations by Woodruff et al. have demonstrated that hepatic involvement most often occurs through diffuse leukemic infiltration rather than through the formation of a discrete mass lesion [16]. Accordingly, presentation of PCL as a mass-forming hepatic lesion is exceedingly rare and may radiographically mimic metastatic carcinoma or primary hepatic malignancies, posing as a potential diagnostic challenge. We report a rare case of PCL presenting as hepatic masses, highlighting its clinicopathologic features and the diagnostic challenges posed by this unusual pattern of organ involvement.

## Case presentation

A 49-year-old man with a past medical history significant for obstructive sleep apnea, gastroesophageal reflux disease, obesity, and chronic back pain presented to the emergency department with several weeks of left-sided rib pain, acute neurological symptoms, and a recent fall. Initial laboratory evaluation revealed anemia and hypercalcemia.

Computed tomography of the abdomen and lumbar spine without intravenous contrast demonstrated an acute to subacute L3 superior endplate compression fracture, chronic compression fractures involving T11, T12, L1, and L2, a 4-cm lobulated soft tissue mass abutting the sacrum, and mild splenomegaly; the liver was otherwise unremarkable at that time, with the exception of a 1.7 cm gallstone in the gallbladder.

Further laboratory evaluation revealed a serum protein electrophoresis demonstrating an M-protein spike of 2.7 g/dL. Serum free light chain analysis showed markedly elevated lambda free light chains at 321 mg/L with suppressed kappa free light chains at 0.5 mg/L, resulting in a kappa-to-lambda ratio of <0.01. Immunoglobulin quantification demonstrated elevated IgG (4,269 mg/dL), IgA (15 mg/dL), and IgM (7 mg/dL). Serum protein immunofixation shows free lambda light chain and IgG lambda monoclonal proteins. Random urine protein electrophoresis was positive for lambda-free light chains, with a total urine protein of 320 mg/dL (Table 1).

**Table 1:** Selected laboratory findings at initial presentation.

Test	Result	Reference Range
Serum Protein Electrophoresis (SPEP)	M-protein spike: 2.7 g/dL	No monoclonal protein present
Serum Free Light Chains - Lambda	321 mg/L	5.7-26.3 mg/L
Serum Free Light Chains - Kappa	0.5 mg/L	3.3-19.4 mg/L
Kappa-to-Lambda Ratio	<0.01	0.26-1.65
Immunoglobulin G (IgG)	4,269 mg/dL	540-1822 mg/dL
Immunoglobulin A (IgA)	15 mg/dL	63-484 mg/dL
Immunoglobulin M (IgM)	7 mg/dL	22-240 mg/dL
Urine Protein Electrophoresis (UPEP)	Positive for lambda free light chains	Negative
Total Urine Protein	320 mg/dL	< 300 mg/24 hours

Bone marrow biopsy showed a hypercellular marrow (approximately 100% cellularity), consisting of 95% lambda-restricted plasma cells. A concurrent peripheral blood smear demonstrated a normochromic, normocytic anemia with 28% circulating plasma cells. Cytogenetic analysis revealed an abnormal clone with a complex karyotype, including loss of chromosome 13. Fluorescence in situ hybridization (FISH) analysis demonstrated deletion of 1p, deletion of 13q, and an IgH/MAF rearrangement consistent with t(14;16). Altogether, these findings were consistent with IgG lambda PCL as defined by both the current WHO 5th edition and the ICC.

**Figure 1:**
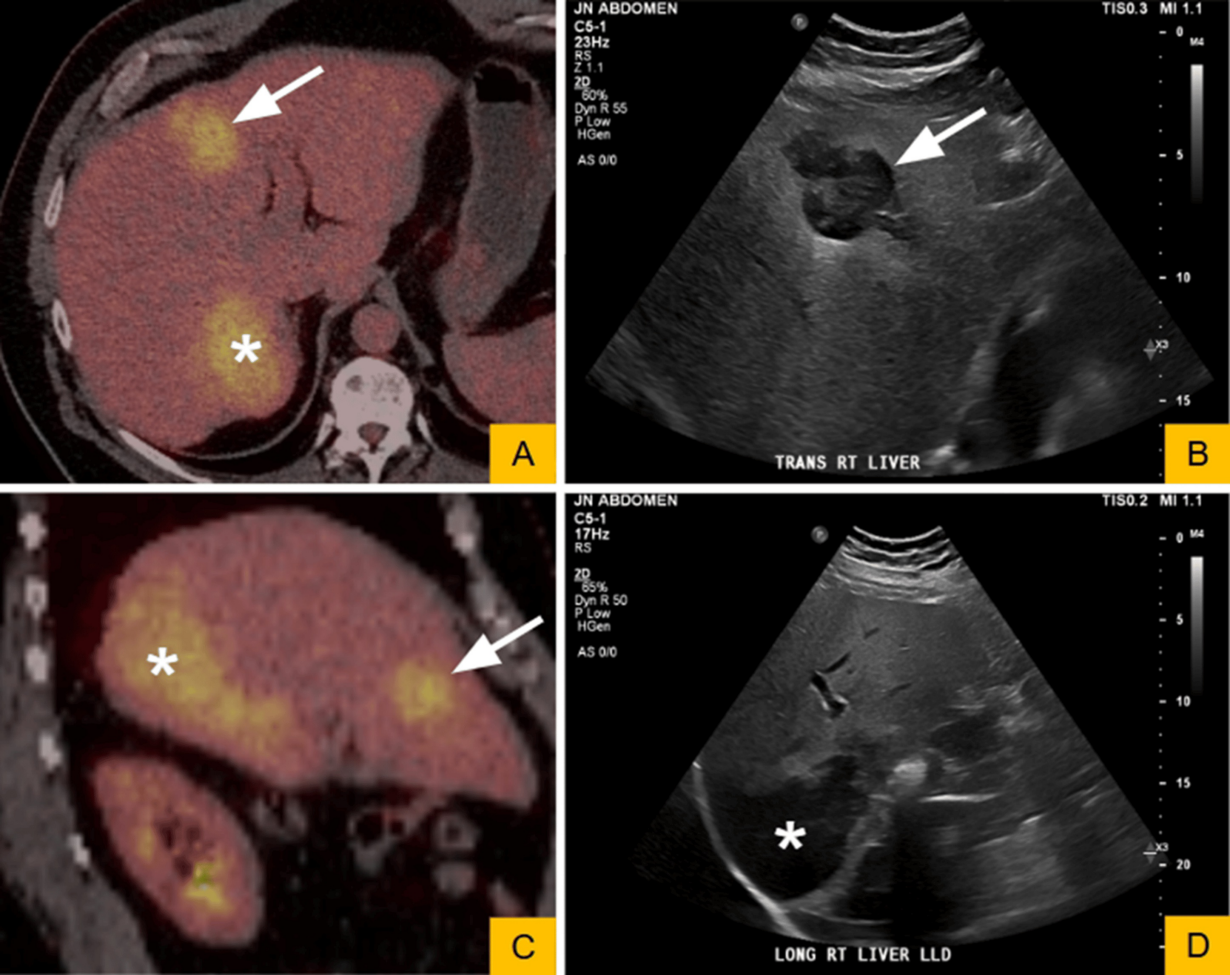
Imaging findings of hepatic involvement by plasma cell leukemia. (A) Axial and (C) sagittal fused fluorodeoxyglucose positron emission tomography (FDG-PET)/non-contrast CT images demonstrate two FDG-avid hepatic masses involving segments 4A/B (arrows) and 7 (asterisks). The segment 7 lesion shows a posterior exophytic component. (B) Corresponding transverse and (D) longitudinal grayscale ultrasound images demonstrate well-circumscribed hypoechoic masses. The anterior lesion in segment 4A/B (arrows) measures up to 5.2 cm, and the posterior segment 7 lesion (asterisks) measures up to 12.2 cm.

The patient was initiated on cyclophosphamide-bortezomib-dexamethasone chemotherapy in combination with daratumumab, with subsequent improvement in symptoms. After three months of chemotherapy, he underwent evaluation for autologous stem cell transplantation. As part of the pre-transplant work-up, 18F-FDG positron emission tomography demonstrated two hypermetabolic lesions within the liver with an SUVmax of 13.4. Of note, liver function tests, including alanine aminotransferase (ALT), aspartate aminotransferase (AST), alkaline phosphatase, and total bilirubin, were within reference ranges at the time of lesion discovery. Follow-up ultrasound a week after confirmed two distinct hepatic lesions, including a well-circumscribed hypoechoic mass measuring 4.3 × 3.9 × 5.2 cm in the anterior left hepatic lobe and a similar-appearing hypoechoic mass measuring 7.4 × 12.2 × 10.6 cm extending to the hepatic capsule in the posterior right hepatic lobe (Figure 1). A percutaneous biopsy of the anterior hepatic lesion was subsequently performed.

**Figure 2:**
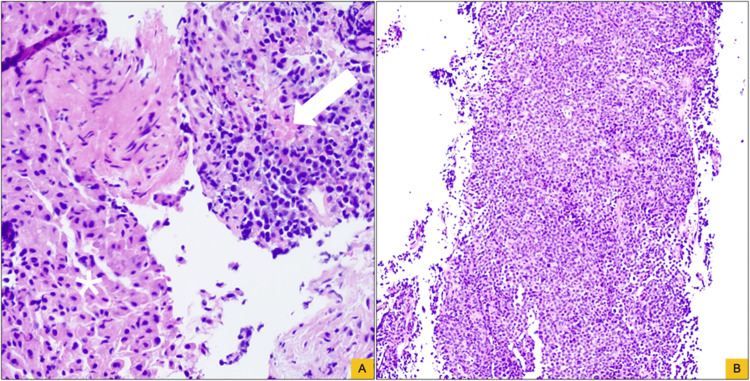
Histologic features of plasma cell leukemia involving the liver. (A) Liver biopsy showing sheets of atypical plasmacytoid cells infiltrating hepatic parenchyma (arrows), with residual hepatocytes at the interface (asterisks), confirming hepatic involvement (hematoxylin and eosin, original magnification ×20). (B) Low-power view demonstrating a dense, mass-forming proliferation of plasma cells arranged in solid sheets, effacing normal hepatic architecture (hematoxylin and eosin, original magnification ×10).

**Figure 3:**
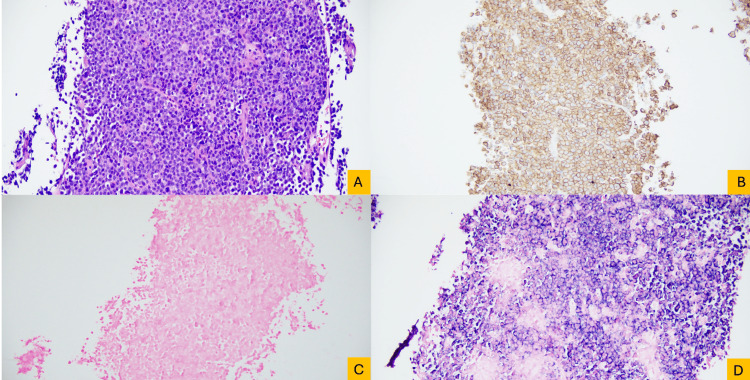
Immunophenotypic confirmation of plasma cell leukemia involving the liver. (A) Liver biopsy showing a solid proliferation of monotonous plasmacytoid cells with eccentric cytoplasm and round to ovoid nuclei exhibiting peripheral chromatin condensation (hematoxylin and eosin, original magnification ×20). (B) Immunohistochemical stain for CD138 highlights strong, diffuse membranous and cytoplasmic positivity in the neoplastic plasma cells (original magnification ×20). (C,D) Light chain in situ hybridization demonstrates light chain restriction, (C) negative staining for kappa, and (D) diffuse cytoplasmic positivity for lambda in the neoplastic cells (original magnification ×20).

Microscopic examination of the liver biopsy revealed a diffuse proliferation of neoplastic cells arranged in solid sheets. The tumor cells demonstrated plasmacytoid morphology, with eccentric nuclei, round-to-ovoid nuclear contours, and moderate cytoplasm. Scattered mitotic figures, single-cell apoptosis, and rare multinucleated “floret-type” cells were identified. Immunohistochemical studies showed strong expression of CD138 and MUM1, with the absence of CAM5.2, CK7, CK20, CD56, and cyclin D1. In situ hybridization demonstrated lambda light chain restriction, confirming mass-forming hepatic involvement by the patient’s known PCL, which is unusual relative to the more frequently reported diffuse/sinusoidal leukemic infiltration of the liver (Figures 2-3).

In light of biopsy-confirmed hepatic involvement by the patient’s PCL despite ongoing therapy, second-line BCMA-directed CAR-T cell therapy (ciltacabtagene autoleucel) was recommended in lieu of the originally planned autologous stem cell transplant. As part of the pre-CAR-T evaluation, a repeat bone marrow assessment demonstrated low-level residual plasma cell neoplasm by flow cytometry. At the time of this case report preparation, the patient is day +21 post-CAR-T therapy and is clinically stable under active follow-up; longer-term response and outcome data are not yet available. 

## Discussion

Plasma cell neoplasms comprise a heterogeneous group of clonal disorders characterized by malignant plasma cell proliferation, most often within the bone marrow, but with variable capacity for dissemination beyond the marrow. The WHO classification categorizes plasma cell neoplasms into plasmacytoma (solitary plasmacytoma of bone and extramedullary plasmacytoma), plasma cell myeloma (including clinical variants such as smoldering myeloma, non-secretory myeloma, and PCL), and plasma cell neoplasms with associated paraneoplastic syndromes (such as polyneuropathy, organomegaly, endocrinopathy, monoclonal plasma cell disorder, and skin changes (POEMS); telangiectasias, elevated erythropoietin level with erythrocytosis, monoclonal gammopathy, perinephric fluid collections, and intrapulmonary shunting (TEMPI); and adenopathy and extensive skin patch overlying a plasmacytoma (AESOP)) [11-15,17].

pPCL is increasingly regarded as a biologically distinct entity [18]. Compared with other plasma cell neoplasms, pPCL tends to present in younger patients and is associated with higher rates of anemia, thrombocytopenia, and hypercalcemia [18]. Cytogenetically, pPCL is enriched for high-risk abnormalities, including t(11;14) and del(17p), whereas hyperdiploidy and certain chromosome 1 abnormalities are more typical of MM [18]. Clinically, pPCL represents the most aggressive end of the plasma cell neoplasm spectrum, characterized by high proliferative activity, rapid disease progression, and dismal prognosis despite modern therapy [3,9-18].

Extramedullary myeloma (EMM) represents an aggressive manifestation of plasma cell myeloma in which a plasma cell clone acquires the capacity to survive and proliferate outside the bone marrow microenvironment [15]. Bladé et al. highlight the heterogeneity of definitions used for extramedullary disease and advocate restricting true EMM to soft-tissue plasmacytomas arising via hematogenous dissemination [15]. PCL is excluded from this definition, as it constitutes a distinct and well-defined pathologic entity [15]. The liver is among the most involved visceral organs in plasma cell neoplasms, although hepatic involvement typically manifests as diffuse infiltration rather than discrete mass lesions [18-22]. In the largest series examining gastrointestinal involvement by plasma cell neoplasms (116 cases from 102 patients), 47% of the cases involved the liver [19]. Notably, mass-forming hepatic lesions are more often associated with EMM than with leukemic disease [15,19].

The present case fulfills diagnostic criteria for pPCL but demonstrates an unusual pattern of hepatic involvement, with discrete mass-forming lesions rather than diffuse hepatomegaly or sinusoidal infiltration. This atypical presentation broadens the morphologic spectrum of hepatic involvement in PCL and raises important diagnostic considerations, including EMM, solitary extramedullary plasmacytoma, metastatic carcinoma, and primary hepatic neoplasms. Careful integration of clinical history, laboratory findings, imaging, and immunophenotypic features is therefore essential.

Extramedullary dissemination in plasma cell neoplasms is associated with high-risk cytogenetic alterations, increased proliferative capacity, apoptosis resistance, and reduced dependence on the bone marrow niche [15]. This was exemplified in our patient whose PCL showed a complex karyotype with deletion of chromosome 13. Experimental models have implicated loss of adhesion molecules, such as VLA-4, in facilitating hematogenous dissemination and extramedullary growth [23]. These mechanisms likely contribute to both leukemic spread and mass-forming extra-marrow involvement in aggressive entities such as PCL.

Biopsy-confirmed hepatic involvement, characterized by negative cytokeratin stains (CAM5.2, CK7, and CK20) and strong expression of plasma cell markers (CD138 and MUM1), established an unusual mass-forming manifestation of PCL and indicated disease progression. Recognition of this atypical pattern of hepatic involvement had direct therapeutic implications, prompting escalation to BCMA-directed cellular therapy rather than upfront autologous stem cell transplantation. Although long-term outcomes remain pending, this case underscores the importance of accurately identifying rare, mass-forming hepatic involvement in PCL, given its diagnostic challenges and potential impact on risk stratification and management.

## Conclusions

In summary, this case highlights a rare presentation of pPCL manifesting as discrete hepatic masses. Recognition of this unusual pattern is critical for accurate diagnosis and appropriate clinical management. This report adds to the limited literature on hepatic mass-forming involvement in PCL and underscores the importance of considering plasma cell neoplasms in the differential diagnosis of hepatic lesions.
